# New polymorph of 2,6-di­methyl­phenol

**DOI:** 10.1107/S2056989026000605

**Published:** 2026-01-29

**Authors:** Thij Slaats, Martin Lutz

**Affiliations:** a Structural Biochemistry, Bijvoet Centre for Biomolecular Research, Faculty of Science, Utrecht University, Universiteitsweg 99, 3584 CG Utrecht, The Netherlands; Katholieke Universiteit Leuven, Belgium

**Keywords:** polymorphism, hy­dro­gen bonding, fingerprint plots, crystal structure, 2,6-dimethylphenol

## Abstract

The racemic monoclinic polymorph of the title com­pound, C_8_H_10_O, is known from the literature [Antona *et al.* (1973 ▸). *Acta Cryst.* B**29**, 1372–1376] and has been redetermined here. Additionally, a new enanti­opure ortho­rhom­bic polymorph is reported. The strongest inter­molecular inter­actions are within one-dimensional hy­dro­gen-bonded chains which are very similar in the two polymorphs. On the other hand, the packing of the chains in the crystal differs significantly between the two forms.

## Abstract 2

The monoclinic and ortho­rhom­bic polymorphs of 2,6-di­methyl­phenol are constituted of very similar hy­dro­gen-bonded chains, but the packing of the chains differs significantly.

## 1. Chemical context

Phenolic com­pounds are important anti­oxidants which occur in biological systems and are present in beverages such as coffee and tea. They are also artificially added to industrial processes to prevent oxidation. Thereby the phenolic hy­droxy group is oxidized to a peroxide and steric strain of substituents at the ring strongly influence this reaction (Drew *et al.*, 1990 ▸). In the medical world, the com­pound 2,6-diiso­propyl­phenol is a relevant drug which is distributed under the name Propofol.

In crystal engineering, phenols belong to the category of bulky alcohols (Brock & Duncan, 1994 ▸). Here, the steric demand of the ring substituents influences the hy­dro­gen-bonding pattern. If the hy­droxy group is the only functional group, it can act both as hy­dro­gen-bond donor and as hy­dro­gen-bond acceptor. For example, unsubstituted phenol in its monoclinic ambient-pressure polymorph forms one-dimensional hy­dro­gen-bonded chains (Zavodnik *et al.*, 1987 ▸). The three independent mol­ecules which constitute the chain are related only by pure translations. The monoclinic high-pressure variant of 2-methyl­phenol again forms one-dimensional hy­dro­gen-bonded chains, but in this structure there is only one independent mol­ecule and the fundamental symmetry operation in the chain is a 2_1_ screw axis along the *b* direction (Oswald & Crichton, 2009 ▸). The corresponding length of the *b* axis is 4.7006 (3) Å. In the crystal structure of 2,6-diiso­propyl­phenol, there are hy­dro­gen-bonded tetra­mers (Bacchi *et al.*, 2016 ▸) and in 2,6-di-*tert*-butyl­phenol, there are no hy­dro­gen bonds (Lutz & Spek, 2005 ▸).

## 2. Structural commentary

The crystal structure of the title com­pound was reported in the literature as monoclinic with the space group *P*2_1_/*c*. Preliminary investigations and unit-cell parameters were reported by Meuthen & von Stackelberg (1960 ▸). A full structure determination by Antona *et al.* (1973 ▸) was based on intensities from film methods [Cambridge Structural Database (CSD; Groom *et al.*, 2016 ▸) refcode DMEPOL10]. In order to improve the quality of the results, we re-investigated this crystal structure with modern equipment and the results are pre­sent­ed here [structure (Ia)]. During our studies, we additionally found a new ortho­rhom­bic polymorph with the space group *P*2_1_2_1_2_1_ [structure (Ib)]. This structure will also be pre­sent­ed and both polymorphs will be com­pared.
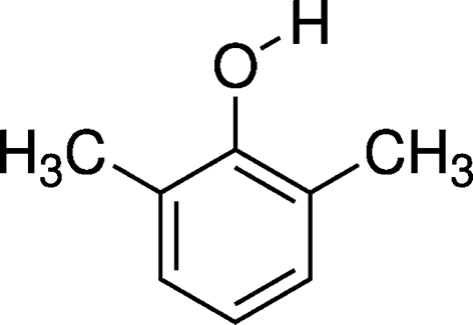


**Figure 1:**
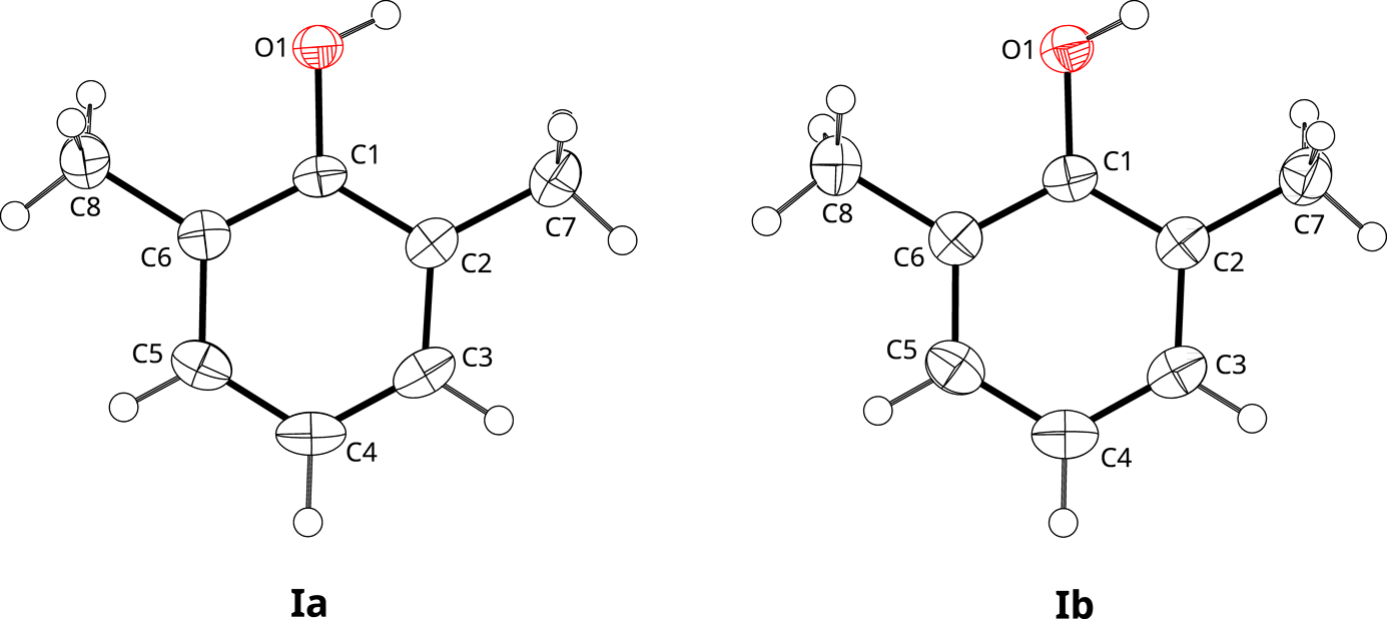
The mol­ecular structures of (Ia) (monoclinic) and (Ib) (ortho­rhom­bic). Displacement ellipsoids are drawn at the 50% probability level and H atoms are drawn with arbitrary radii.

The mol­ecular structures of the monoclinic (Ia) and orthorhom­bic (Ib) polymorphs are very similar and differ only in the conformations of the methyl groups (Fig. 1 ▸). There are no significant differences in the bond lengths and angles between the two structures (Tables 1 ▸ and 2 ▸). The mol­ecules are essentially planar, with a maximum deviation from the plane of 0.0330 (9) Å for atom O1 in (Ia) and 0.0158 (17) Å for atom C1 in (Ib). Consequently, the non-H atoms of the mol­ecule have approximate *C*_2*v*_ symmetry, with an r.m.s. deviation of 0.0754 Å for (Ia) and 0.0725 Å for (Ib).

## 3. Supra­molecular features

**Figure 2:**
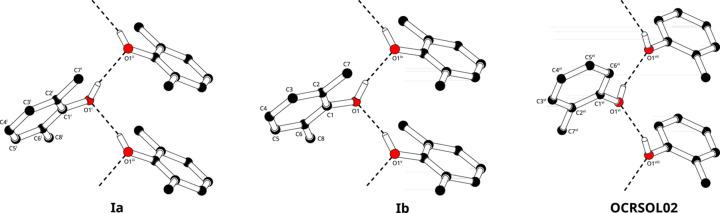
Hydrogen-bonded one-dimensional chains. C—H hy­dro­gens are omitted for clarity. For (Ia), the *b* axis is oriented verically in the plane of the drawing. [Symmetry codes: (i) −*x* + 1, −*y* + 1, −*z* + 1; (ii) *x* + 1, −*y* + 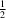, *z* + 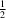; (iii) *x* + 1, −*y* + 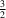, *z* + 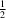.] For (Ib), the *a* axis is oriented verically in the plane of the drawing. [Symmetry codes: (iv) *x* + 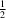, −*y* + 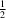, −*z* + 2; (v) *x* − 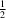, −*y* + 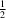, −*z* + 2.] For 2-methyl­phenol (CSD refcode OCRSOL02; Oswald & Crichton, 2009 ▸), the *b* axis is oriented verically in the plane of the drawing. [Symmetry codes: (vi) −*x* + 1, −*y* + 1, −*z* + 1; (vii) *x* + 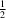, −*y* + 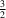, *z* − 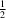; (viii) *x* + 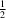, −*y* + 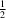, *z* − 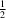.]

The hy­dro­gen-bonding patterns in (Ia) and (Ib) are very similar (Fig. 2 ▸, and Tables 3 ▸ and 4 ▸). The mol­ecules form one-dimensional chains, in (Ia) along the *b* axis and in (Ib) along the *a* axis. In both cases, the symmetry operation relating the mol­ecules within one chain is a 2_1_ screw axis. In (Ia), the unit-cell length of the *b* axis is 4.45179 (19) Å and in (Ib) the *a* axis is 4.3981 (4) Å. By the screw symmetry, each individual chain is helically chiral. In centrosymmetric (Ia), the overall structure is racemic, and in (Ib) the overall structure is enanti­opure. The absolute structure of (Ib) could not be determined reliably from anomalous scattering. The one-dimensional chains are com­parable with racemic 2-methyl­phenol (Oswald & Crichton, 2009 ▸; CSD refcode OCRSOL02). While the O—H⋯O geometry in the three structures is similar, the arrangement of the mol­ecular planes differs slightly. In (Ia), the mol­ecular plane has an angle of 53.11 (3)° with the *b* axis, in (Ib) there is an angle of 55.58 (4)° with the *a* axis and in 2-methyl­phenol there is an angle of 47.20 (11)° with the *b* axis.

As a consequence of the hy­dro­gen-bonding scheme, the C—C—O—H torsion angles are similar: 20.2 (13)° in racemic (Ia) and −30.0 (18)° in (Ib). This is in contrast to 2,6-di-*tert*-butyl­phenol (Lutz & Spek, 2005 ▸) which does not form hy­dro­gen bonds and where the hy­droxy group is in the mol­ecular plane.

In addition to the hy­dro­gen bonding, there are weak π–π stacking inter­actions within the chains, *i.e.* along the *b* direction in (Ia) and along the *a* direction in (Ib). The corresponding symmetry operations are pure translations (Table 5 ▸). Consequently, the involved rings are exactly parallel, but because the rings are tilted with respect to the crystallographic axes, respectively, the ring slippage is rather large.

**Figure 3:**
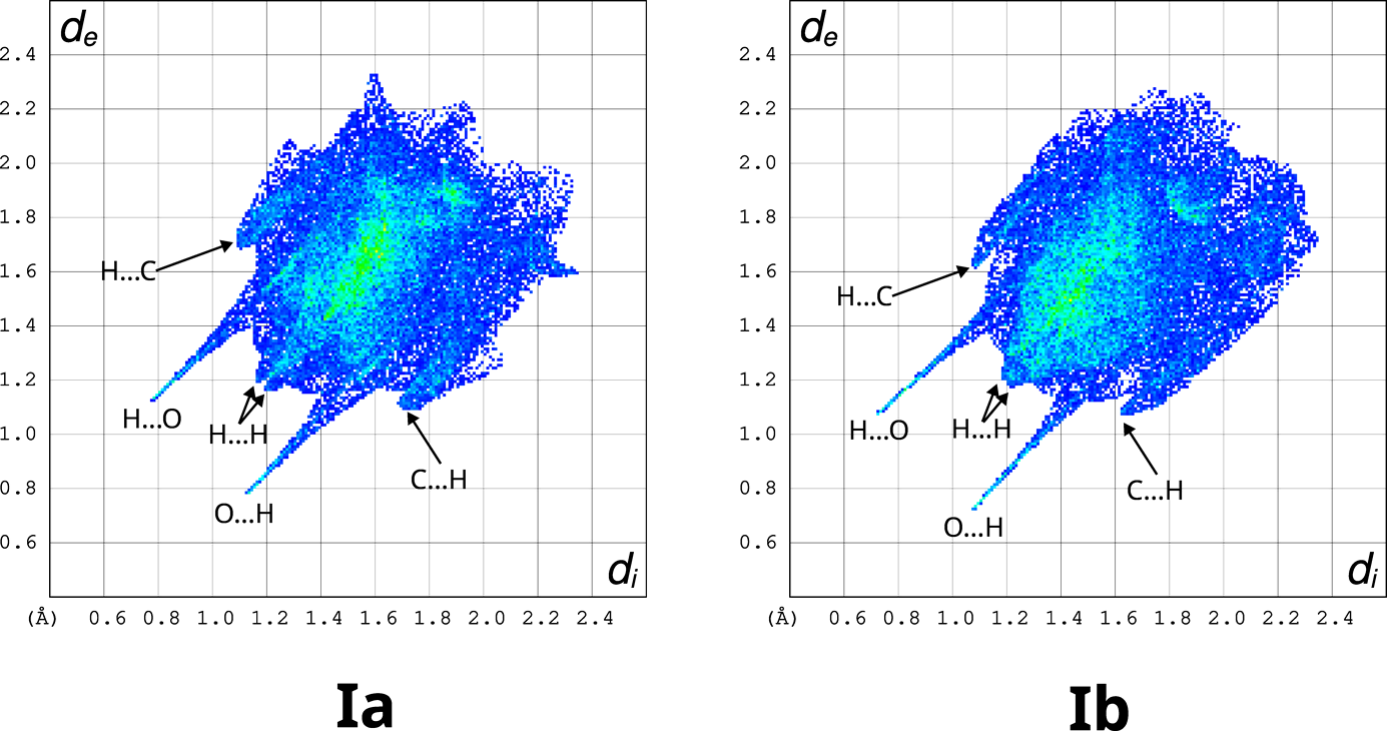
Hirshfeld surface fingerprint plots (McKinnon *et al.*, 2004 ▸) for (Ia) and (Ib) prepared with *CrystalExplorer* (Version 21.5; Spackman *et al.*, 2021 ▸).

The geometrical analysis of inter­molecular inter­actions is confirmed by the calculation of Hirshfeld surface fingerprint plots (McKinnon *et al.*, 2004 ▸) for (Ia) and (Ib), which show a high similarity between the two polymorphs (Fig. 3 ▸). This similarity is a strong indication that all major inter­molecular bonds are within the hy­dro­gen-bonded chains. Enrichment ratios (Jelsch *et al.*, 2014 ▸) derived from the fingerprint plots (Tables 6 ▸ and 7 ▸) highlight the propensity for H⋯O and C⋯C inter­actions, *i.e.* hy­dro­gen bonds and π–π stacking.

**Figure 4:**
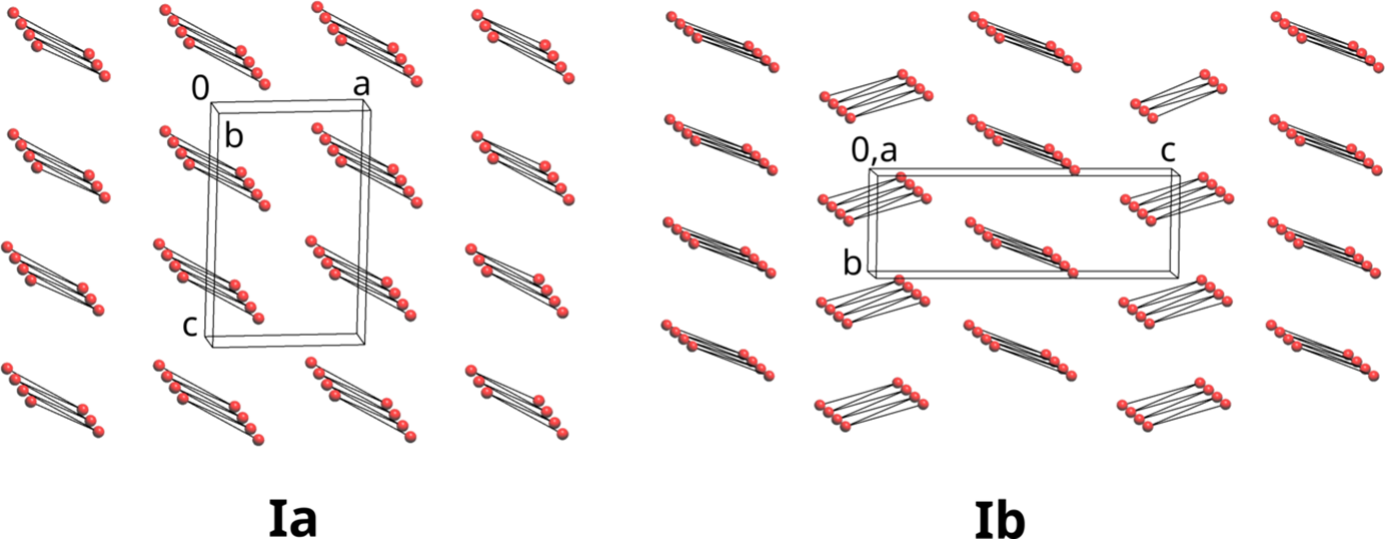
Simplified structures of (Ia) and (Ib). Red spheres are the simplified individual mol­ecules of 2,6-di­methyl­phenol and the connecting lines are the O—H⋯O hy­dro­gen bonds. Simplification and plot preparation was done with *ToposPro* (Blatov *et al.*, 2014 ▸).

While the geometry within the hy­dro­gen-bonded chains in (Ia) and (Ib) is very similar, the packing of the chains is significantly different (Fig. 4 ▸). The inversion centres between the chains in (Ia) result in the coplanarity of the rings in adjacent chains. The chains in (Ib) are related to each other by 2_1_ screw axes, resulting in a dihedral angle of 50.70° between the planes of the rings in adjacent chains.

Despite the different packing of the chains, the crystal density in (Ia) and (Ib) is very similar with values of 1.1754 (1) and 1.1819 (1) g cm^3^, respectively. The corresponding packing indices (Kitajgorodskij, 1973 ▸) of 68.4% for (Ia) and 69.0% for (Ib) are consistent with this. Based on this information, it cannot be decided which of the two polymorphs is more stable.

## 4. Synthesis and crystallization

Commercial 2,6-di­methyl­phenol (Sigma–Aldrich) was used as starting material. Crystals of (Ia) were obtained by slow evaporation from a solution in hexane. Crystals of (Ib) were obtained by slow evaporation from a solution in ethanol. Note that the hexane solution gave crystals of both forms. Both crystal forms are very brittle and difficult to cut.

## 5. Refinement

Crystal data, data collection and structure refinement details are summarized in Table 8 ▸. The intensity integration of (Ia) involved a large isotropic mosaicity of 1.3° for the prediction of the reflection profiles. For (Ib), an isotropic mosaicity of 1.5° plus an anisotropic mosaicity of 0.45° about *hkl*=(0,0,1) was involved.

In the refinements of (Ia) and (Ib), O—H hy­dro­gens were refined freely with isotropic displacement parameters and C—H hy­dro­gens were refined with a riding model.

## Supplementary Material

Crystal structure: contains datablock(s) Ia, Ib, global. DOI: 10.1107/S2056989026000605/vm2324sup1.cif

Structure factors: contains datablock(s) Ia. DOI: 10.1107/S2056989026000605/vm2324Iasup2.hkl

Structure factors: contains datablock(s) Ib. DOI: 10.1107/S2056989026000605/vm2324Ibsup3.hkl

Supporting information file. DOI: 10.1107/S2056989026000605/vm2324Iasup4.cml

Supporting information file. DOI: 10.1107/S2056989026000605/vm2324Ibsup5.cml

CCDC references: 2524770, 2524769

Additional supporting information:  crystallographic information; 3D view; checkCIF report

## Figures and Tables

**Table 1:** Comparison of bond lengths (Å) in (Ia) and (Ib)

	(Ia)	(Ib)	Δ	Δ/σ
O1—C1	1.3862 (16)	1.3913 (19)	−0.0051 (25)	−2.04
C1—C6	1.393 (2)	1.392 (2)	0.0010 (28)	0.36
C1—C2	1.3958 (19)	1.398 (2)	−0.0022 (28)	−0.79
C2—C3	1.3892 (19)	1.389 (2)	0.0002 (28)	0.07
C2—C7	1.496 (2)	1.503 (3)	−0.007 (4)	−1.75
C3—C4	1.378 (2)	1.380 (3)	−0.002 (4)	−0.50
C4—C5	1.382 (2)	1.385 (3)	−0.003 (4)	−0.75
C5—C6	1.389 (2)	1.387 (2)	0.0020 (28)	0.71
C6—C8	1.5068 (19)	1.509 (3)	−0.002 (4)	−0.50

**Table 2:** Comparison of bond angles (°) in (Ia) and (Ib)

	(Ia)	(Ib)	Δ	Δ/σ
O1—C1—C6	116.06 (12)	116.36 (15)	−0.30 (19)	−1.58
O1—C1—C2	121.67 (13)	121.28 (16)	0.39 (21)	1.85
C6—C1—C2	122.24 (12)	122.32 (16)	−0.08 (20)	−0.40
C3—C2—C1	117.39 (14)	117.38 (17)	0.01 (22)	0.05
C3—C2—C7	121.12 (13)	120.93 (17)	0.19 (21)	0.90
C1—C2—C7	121.50 (12)	121.69 (16)	−0.19 (20)	−0.95
C4—C3—C2	121.72 (14)	121.58 (18)	0.14 (23)	0.61
C3—C4—C5	119.59 (14)	119.64 (18)	−0.05 (23)	−0.22
C4—C5—C6	121.03 (15)	121.01 (19)	0.02 (24)	0.08
C5—C6—C1	118.03 (13)	118.05 (16)	−0.02 (21)	−0.10
C5—C6—C8	120.96 (14)	121.06 (17)	−0.10 (22)	−0.45
C1—C6—C8	121.01 (12)	120.88 (16)	0.13 (20)	0.65

**Table 3:** Hydrogen-bond geometry (Å, °) for (Ia)

*D*—H⋯*A*	*D*—H	H⋯*A*	*D*⋯*A*	*D*—H⋯*A*
O1—H1⋯O1^i^	0.840 (19)	2.032 (18)	2.8087 (12)	153.4 (15)

Symmetry code: (i) 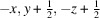.

**Table 4:** Hydrogen-bond geometry (Å, °) for (Ib)

*D*—H⋯*A*	*D*—H	H⋯*A*	*D*⋯*A*	*D*—H⋯*A*
O1—H1⋯O1^i^	0.90 (3)	1.89 (3)	2.7470 (14)	158 (2)

Symmetry code: (i) 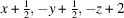.

**Table 5:** Weak π–π stacking in (Ia) and (Ib) *Cg* stands for center of gravity.

Structure	Ring⋯ring	Perpendicular ring–ring distance (Å)	*Cg*⋯*Cg* distance (Å)	Slippage (Å)
(Ia)	C1–C6⋯C1–C6^i^	3.5387 (6)	4.4518 (9)	2.701
(Ib)	C1–C6⋯C1–C6^ii^	3.6085 (8)	4.3981 (12)	2.514

Symmetry codes: (i) *x*, *y* + 1, *z*; (ii) *x* + 1, *y*, *z*.

**Table 6:** Enrichment ratios for (Ia) calculated by the approach of Jelsch *et al.* (2014 ▸) from the Hirshfeld surface fingerprint plot

	H	C	O
H	1.00	–	–
C	0.94	1.78	–
O	1.22	–	–

**Table 7:** Enrichment ratios for (Ib) calculated by the approach of Jelsch *et al.* (2014 ▸) from the Hirshfeld surface fingerprint plot

	H	C	O
H	1.00	–	–
C	0.91	1.95	–
O	1.21	–	–

**Table 8:** Experimental details For both structures: C_8_H_10_O, *M*_r_ = 122.16, *Z* = 4. Experiments were carried out at 150 K with Mo *K*α radiation using a Bruker Kappa APEXII area-detector diffractometer. Absorption was corrected for by multi-scan methods (*SADABS2016*; Krause *et al.*, 2015 ▸). Refinement was on 88 parameters. H atoms were treated by a mixture of independent and constrained refinement.

	(Ia)	(Ib)
Crystal data		
Crystal system, space group	Monoclinic, *P*2_1_/*c*	Orthorhombic, *P*2_1_2_1_2_1_
*a*, *b*, *c* (Å)	10.0160 (5), 4.45179 (19), 15.4874 (7)	4.3981 (4), 7.2646 (4), 21.4884 (12)
α, β, γ (°)	90, 91.533 (3), 90	90, 90, 90
*V* (Å^3^)	690.32 (6)	686.56 (8)
μ (mm^−1^)	0.08	0.08
Crystal size (mm)	0.27 × 0.10 × 0.05	0.29 × 0.13 × 0.08

Data collection		
*T*_min_, *T*_max_	0.635, 0.746	0.572, 0.746
No. of measured, independent and observed [*I* > 2σ(*I*)] reflections	12932, 1585, 1058	9552, 1583, 1372
*R*_int_	0.054	0.030
(sin θ/λ)_max_ (Å^−1^)	0.650	0.650

Refinement		
*R*[*F*^2^ > 2σ(*F*^2^)], *wR*(*F*^2^), *S*	0.044, 0.106, 1.04	0.038, 0.089, 1.06
No. of reflections	1585	1583
Δρ_max_, Δρ_min_ (e Å^−3^)	0.19, −0.16	0.12, −0.15
Absolute structure	–	Flack *x* determined using 493 quotients [(*I*^+^) − (*I*^−^)]/[(*I*^+^) + (*I*^−^)] (Parsons *et al.*, 2013 ▸)
Absolute structure parameter	–	0.2 (6)

Computer programs: *APEX3* (Bruker, 2012 ▸), *PEAKREF* (Schreurs, 2016 ▸), *Eval15* (Schreurs *et al.*, 2010 ▸), *SADABS2016* (Krause *et al.*, 2015 ▸), *SHELXS* (Sheldrick, 2008 ▸), *SHELXL2019* (Sheldrick, 2015 ▸), *PLATON* (Spek, 2020 ▸), *CrystalExplorer* (Spackman *et al.*, 2021 ▸) and *ToposPro* (Blatov *et al.*, 2014 ▸).

## supplementary crystallographic information

### 2,6-Dimethylphenol (Ia) Crystal data

**Table:**

C_8_H_10_O	*F*(000) = 264
*M**_r_* = 122.16	*D*_x_ = 1.175 Mg m^−^^3^
Monoclinic, *P*2_1_/*c*	Mo *K*α radiation, λ = 0.71073 Å
*a* = 10.0160 (5) Å	Cell parameters from 5797 reflections
*b* = 4.45179 (19) Å	θ = 1.3–27.5°
*c* = 15.4874 (7) Å	µ = 0.08 mm^−^^1^
β = 91.533 (3)°	*T* = 150 K
*V* = 690.32 (6) Å^3^	Needle, colourless
*Z* = 4	0.27 × 0.10 × 0.05 mm

### 2,6-Dimethylphenol (Ia) Data collection

**Table:**

Bruker Kappa APEXII area detector diffractometer	1058 reflections with *I* > 2σ(*I*)
Radiation source: sealed tube	*R*_int_ = 0.054
φ and ω scans	θ_max_ = 27.5°, θ_min_ = 2.0°
Absorption correction: multi-scan (SADABS2016; Krause *et al.*, 2015)	*h* = −13→13
*T*_min_ = 0.635, *T*_max_ = 0.746	*k* = −5→5
12932 measured reflections	*l* = −20→20
1585 independent reflections	

### 2,6-Dimethylphenol (Ia) Refinement

**Table:**

Refinement on *F*^2^	Primary atom site location: structure-invariant direct methods
Least-squares matrix: full	Secondary atom site location: difference Fourier map
*R*[*F*^2^ > 2σ(*F*^2^)] = 0.044	Hydrogen site location: difference Fourier map
*wR*(*F*^2^) = 0.106	H atoms treated by a mixture of independent and constrained refinement
*S* = 1.04	*w* = 1/[σ^2^(*F*_o_^2^) + (0.041*P*)^2^ + 0.1557*P*] where *P* = (*F*_o_^2^ + 2*F*_c_^2^)/3
1585 reflections	(Δ/σ)_max_ < 0.001
88 parameters	Δρ_max_ = 0.19 e Å^−^^3^
0 restraints	Δρ_min_ = −0.16 e Å^−^^3^

### 2,6-Dimethylphenol (Ia) Special details

**Table:**

Geometry. All esds (except the esd in the dihedral angle between two l.s. planes) are estimated using the full covariance matrix. The cell esds are taken into account individually in the estimation of esds in distances, angles and torsion angles; correlations between esds in cell parameters are only used when they are defined by crystal symmetry. An approximate (isotropic) treatment of cell esds is used for estimating esds involving l.s. planes.

### 2,6-Dimethylphenol (Ia) Fractional atomic coordinates and isotropic or equivalent isotropic displacement parameters (Å^2^)

**Table:**

	*x*	*y*	*z*	*U*_iso_*/*U*_eq_	
O1	0.08542 (10)	0.5797 (2)	0.24855 (6)	0.0284 (3)	
H1	0.0445 (17)	0.732 (4)	0.2661 (11)	0.048 (6)*	
C1	0.18926 (13)	0.4937 (3)	0.30414 (9)	0.0245 (3)	
C2	0.19468 (13)	0.5837 (3)	0.39048 (9)	0.0273 (3)	
C3	0.30044 (15)	0.4779 (4)	0.44177 (9)	0.0348 (4)	
H3	0.307081	0.537423	0.500640	0.042*	
C4	0.39598 (15)	0.2888 (4)	0.40949 (10)	0.0390 (4)	
H4	0.466787	0.217345	0.445997	0.047*	
C5	0.38813 (14)	0.2039 (4)	0.32369 (10)	0.0358 (4)	
H5	0.454183	0.073940	0.301499	0.043*	
C6	0.28515 (14)	0.3056 (3)	0.26936 (9)	0.0289 (3)	
C7	0.09068 (16)	0.7856 (4)	0.42675 (9)	0.0356 (4)	
H7A	0.088815	0.975694	0.394838	0.053*	
H7B	0.112039	0.824889	0.487829	0.053*	
H7C	0.003074	0.688351	0.421333	0.053*	
C8	0.27789 (15)	0.2164 (4)	0.17549 (9)	0.0375 (4)	
H8A	0.205148	0.071901	0.166053	0.056*	
H8B	0.362625	0.124388	0.159553	0.056*	
H8C	0.261160	0.395016	0.139823	0.056*	

### 2,6-Dimethylphenol (Ia) Atomic displacement parameters (Å^2^)

**Table:**

	*U*^11^	*U*^22^	*U*^33^	*U*^12^	*U*^13^	*U*^23^
O1	0.0301 (6)	0.0258 (6)	0.0289 (6)	0.0026 (5)	−0.0060 (4)	−0.0013 (5)
C1	0.0239 (7)	0.0218 (7)	0.0275 (7)	−0.0058 (6)	−0.0050 (6)	0.0045 (6)
C2	0.0306 (7)	0.0251 (8)	0.0261 (7)	−0.0082 (6)	0.0000 (6)	0.0028 (6)
C3	0.0399 (8)	0.0370 (9)	0.0271 (8)	−0.0116 (7)	−0.0075 (7)	0.0049 (7)
C4	0.0306 (8)	0.0420 (10)	0.0436 (9)	−0.0031 (7)	−0.0123 (7)	0.0109 (8)
C5	0.0269 (7)	0.0347 (9)	0.0457 (9)	0.0015 (7)	−0.0019 (7)	0.0040 (7)
C6	0.0272 (7)	0.0277 (8)	0.0317 (8)	−0.0046 (6)	0.0004 (6)	0.0015 (6)
C7	0.0439 (9)	0.0356 (9)	0.0275 (8)	−0.0030 (7)	0.0017 (6)	−0.0012 (7)
C8	0.0344 (8)	0.0420 (10)	0.0363 (9)	0.0036 (7)	0.0020 (7)	−0.0072 (7)

### 2,6-Dimethylphenol (Ia) Geometric parameters (Å, º)

**Table:**

O1—C1	1.3862 (16)	C5—C6	1.389 (2)
O1—H1	0.840 (19)	C5—H5	0.9500
C1—C6	1.393 (2)	C6—C8	1.5068 (19)
C1—C2	1.3958 (19)	C7—H7A	0.9800
C2—C3	1.3892 (19)	C7—H7B	0.9800
C2—C7	1.496 (2)	C7—H7C	0.9800
C3—C4	1.378 (2)	C8—H8A	0.9800
C3—H3	0.9500	C8—H8B	0.9800
C4—C5	1.382 (2)	C8—H8C	0.9800
C4—H4	0.9500		
			
C1—O1—H1	112.7 (11)	C5—C6—C1	118.03 (13)
O1—C1—C6	116.06 (12)	C5—C6—C8	120.96 (14)
O1—C1—C2	121.67 (13)	C1—C6—C8	121.01 (12)
C6—C1—C2	122.24 (12)	C2—C7—H7A	109.5
C3—C2—C1	117.39 (14)	C2—C7—H7B	109.5
C3—C2—C7	121.12 (13)	H7A—C7—H7B	109.5
C1—C2—C7	121.50 (12)	C2—C7—H7C	109.5
C4—C3—C2	121.72 (14)	H7A—C7—H7C	109.5
C4—C3—H3	119.1	H7B—C7—H7C	109.5
C2—C3—H3	119.1	C6—C8—H8A	109.5
C3—C4—C5	119.59 (14)	C6—C8—H8B	109.5
C3—C4—H4	120.2	H8A—C8—H8B	109.5
C5—C4—H4	120.2	C6—C8—H8C	109.5
C4—C5—C6	121.03 (15)	H8A—C8—H8C	109.5
C4—C5—H5	119.5	H8B—C8—H8C	109.5
C6—C5—H5	119.5		
			
O1—C1—C2—C3	177.94 (12)	C3—C4—C5—C6	−0.2 (2)
C6—C1—C2—C3	−0.1 (2)	C4—C5—C6—C1	−0.6 (2)
O1—C1—C2—C7	−1.9 (2)	C4—C5—C6—C8	179.08 (14)
C6—C1—C2—C7	−179.92 (13)	O1—C1—C6—C5	−177.45 (12)
C1—C2—C3—C4	−0.7 (2)	C2—C1—C6—C5	0.7 (2)
C7—C2—C3—C4	179.16 (14)	O1—C1—C6—C8	2.9 (2)
C2—C3—C4—C5	0.8 (2)	C2—C1—C6—C8	−178.95 (13)

### 2,6-Dimethylphenol (Ia) Hydrogen-bond geometry (Å, º)

**Table:**

*D*—H···*A*	*D*—H	H···*A*	*D*···*A*	*D*—H···*A*
O1—H1···O1^i^	0.840 (19)	2.032 (18)	2.8087 (12)	153.4 (15)

Symmetry code: (i) −*x*, *y*+1/2, −*z*+1/2.

### 2,6-Dimethylphenol (Ib) Crystal data

**Table:**

C_8_H_10_O	*D*_x_ = 1.182 Mg m^−^^3^
*M**_r_* = 122.16	Mo *K*α radiation, λ = 0.71073 Å
Orthorhombic, *P*2_1_2_1_2_1_	Cell parameters from 6677 reflections
*a* = 4.3981 (4) Å	θ = 1.9–27.6°
*b* = 7.2646 (4) Å	µ = 0.08 mm^−^^1^
*c* = 21.4884 (12) Å	*T* = 150 K
*V* = 686.56 (8) Å^3^	Needle, colourless
*Z* = 4	0.29 × 0.13 × 0.08 mm
*F*(000) = 264	

### 2,6-Dimethylphenol (Ib) Data collection

**Table:**

Bruker Kappa APEXII area detector diffractometer	1372 reflections with *I* > 2σ(*I*)
Radiation source: sealed tube	*R*_int_ = 0.030
φ and ω scans	θ_max_ = 27.5°, θ_min_ = 1.9°
Absorption correction: multi-scan (SADABS2016; Krause *et al.*, 2015)	*h* = −5→5
*T*_min_ = 0.572, *T*_max_ = 0.746	*k* = −9→9
9552 measured reflections	*l* = −27→27
1583 independent reflections	

### 2,6-Dimethylphenol (Ib) Refinement

**Table:**

Refinement on *F*^2^	Secondary atom site location: difference Fourier map
Least-squares matrix: full	Hydrogen site location: difference Fourier map
*R*[*F*^2^ > 2σ(*F*^2^)] = 0.038	H atoms treated by a mixture of independent and constrained refinement
*wR*(*F*^2^) = 0.089	*w* = 1/[σ^2^(*F*_o_^2^) + (0.0382*P*)^2^ + 0.1305*P*] where *P* = (*F*_o_^2^ + 2*F*_c_^2^)/3
*S* = 1.06	(Δ/σ)_max_ < 0.001
1583 reflections	Δρ_max_ = 0.12 e Å^−^^3^
88 parameters	Δρ_min_ = −0.15 e Å^−^^3^
0 restraints	Absolute structure: Flack *x* determined using 493 quotients [(I+)-(I-)]/[(I+)+(I-)] (Parsons *et al.*, 2013)
Primary atom site location: structure-invariant direct methods	Absolute structure parameter: 0.2 (6)

### 2,6-Dimethylphenol (Ib) Special details

**Table:**

Geometry. All esds (except the esd in the dihedral angle between two l.s. planes) are estimated using the full covariance matrix. The cell esds are taken into account individually in the estimation of esds in distances, angles and torsion angles; correlations between esds in cell parameters are only used when they are defined by crystal symmetry. An approximate (isotropic) treatment of cell esds is used for estimating esds involving l.s. planes.

### 2,6-Dimethylphenol (Ib) Fractional atomic coordinates and isotropic or equivalent isotropic displacement parameters (Å^2^)

**Table:**

	*x*	*y*	*z*	*U*_iso_*/*U*_eq_	
O1	0.0115 (3)	0.24139 (17)	0.96180 (5)	0.0332 (3)	
H1	0.188 (6)	0.271 (4)	0.9805 (10)	0.056 (7)*	
C1	−0.0283 (4)	0.3341 (2)	0.90574 (7)	0.0282 (4)	
C2	0.0962 (4)	0.5089 (2)	0.89606 (8)	0.0305 (4)	
C3	0.0425 (5)	0.5920 (3)	0.83892 (8)	0.0362 (5)	
H3	0.128018	0.709627	0.830709	0.043*	
C4	−0.1321 (5)	0.5079 (3)	0.79378 (9)	0.0408 (5)	
H4	−0.166637	0.567537	0.755069	0.049*	
C5	−0.2567 (5)	0.3362 (3)	0.80521 (8)	0.0379 (5)	
H5	−0.378602	0.279108	0.774230	0.046*	
C6	−0.2063 (4)	0.2462 (3)	0.86119 (8)	0.0314 (4)	
C7	0.2806 (5)	0.6043 (3)	0.94534 (9)	0.0383 (5)	
H7A	0.476881	0.541912	0.949973	0.058*	
H7B	0.170690	0.600535	0.984991	0.058*	
H7C	0.314514	0.732705	0.933190	0.058*	
C8	−0.3386 (5)	0.0581 (3)	0.87339 (9)	0.0400 (5)	
H8A	−0.506899	0.068676	0.903074	0.060*	
H8B	−0.181070	−0.022347	0.890737	0.060*	
H8C	−0.413907	0.005669	0.834295	0.060*	

### 2,6-Dimethylphenol (Ib) Atomic displacement parameters (Å^2^)

**Table:**

	*U*^11^	*U*^22^	*U*^33^	*U*^12^	*U*^13^	*U*^23^
O1	0.0344 (8)	0.0349 (6)	0.0305 (6)	−0.0030 (7)	−0.0010 (6)	0.0056 (5)
C1	0.0286 (9)	0.0295 (8)	0.0264 (8)	0.0054 (8)	0.0056 (7)	0.0014 (7)
C2	0.0301 (9)	0.0286 (8)	0.0328 (9)	0.0040 (8)	0.0060 (7)	−0.0008 (7)
C3	0.0393 (11)	0.0326 (9)	0.0367 (9)	0.0033 (9)	0.0070 (9)	0.0053 (8)
C4	0.0439 (12)	0.0461 (11)	0.0323 (9)	0.0065 (11)	0.0020 (9)	0.0080 (8)
C5	0.0378 (11)	0.0461 (11)	0.0299 (9)	0.0027 (10)	−0.0009 (8)	−0.0031 (8)
C6	0.0304 (9)	0.0317 (9)	0.0320 (9)	0.0036 (9)	0.0048 (7)	−0.0028 (7)
C7	0.0430 (12)	0.0316 (9)	0.0404 (10)	−0.0034 (9)	0.0020 (9)	−0.0008 (8)
C8	0.0439 (12)	0.0344 (10)	0.0418 (10)	−0.0043 (9)	−0.0022 (10)	−0.0037 (8)

### 2,6-Dimethylphenol (Ib) Geometric parameters (Å, º)

**Table:**

O1—C1	1.3913 (19)	C5—C6	1.387 (2)
O1—H1	0.90 (3)	C5—H5	0.9500
C1—C6	1.392 (2)	C6—C8	1.509 (3)
C1—C2	1.398 (2)	C7—H7A	0.9800
C2—C3	1.389 (2)	C7—H7B	0.9800
C2—C7	1.503 (3)	C7—H7C	0.9800
C3—C4	1.380 (3)	C8—H8A	0.9800
C3—H3	0.9500	C8—H8B	0.9800
C4—C5	1.385 (3)	C8—H8C	0.9800
C4—H4	0.9500		
			
C1—O1—H1	112.2 (15)	C5—C6—C1	118.05 (16)
O1—C1—C6	116.36 (15)	C5—C6—C8	121.06 (17)
O1—C1—C2	121.28 (16)	C1—C6—C8	120.88 (16)
C6—C1—C2	122.32 (16)	C2—C7—H7A	109.5
C3—C2—C1	117.38 (17)	C2—C7—H7B	109.5
C3—C2—C7	120.93 (17)	H7A—C7—H7B	109.5
C1—C2—C7	121.69 (16)	C2—C7—H7C	109.5
C4—C3—C2	121.58 (18)	H7A—C7—H7C	109.5
C4—C3—H3	119.2	H7B—C7—H7C	109.5
C2—C3—H3	119.2	C6—C8—H8A	109.5
C3—C4—C5	119.64 (18)	C6—C8—H8B	109.5
C3—C4—H4	120.2	H8A—C8—H8B	109.5
C5—C4—H4	120.2	C6—C8—H8C	109.5
C4—C5—C6	121.01 (19)	H8A—C8—H8C	109.5
C4—C5—H5	119.5	H8B—C8—H8C	109.5
C6—C5—H5	119.5		
			
O1—C1—C2—C3	−179.36 (17)	C3—C4—C5—C6	−0.7 (3)
C6—C1—C2—C3	−1.6 (2)	C4—C5—C6—C1	0.4 (3)
O1—C1—C2—C7	0.5 (3)	C4—C5—C6—C8	−179.23 (19)
C6—C1—C2—C7	178.23 (18)	O1—C1—C6—C5	178.60 (16)
C1—C2—C3—C4	1.4 (3)	C2—C1—C6—C5	0.8 (3)
C7—C2—C3—C4	−178.49 (18)	O1—C1—C6—C8	−1.8 (2)
C2—C3—C4—C5	−0.3 (3)	C2—C1—C6—C8	−179.61 (17)

### 2,6-Dimethylphenol (Ib) Hydrogen-bond geometry (Å, º)

**Table:**

*D*—H···*A*	*D*—H	H···*A*	*D*···*A*	*D*—H···*A*
O1—H1···O1^i^	0.90 (3)	1.89 (3)	2.7470 (14)	158 (2)

Symmetry code: (i) *x*+1/2, −*y*+1/2, −*z*+2.
